# Effectiveness of an Internet-Based, Self-Guided, Short-Term Mindfulness Training (ISSMT) Program for Relieving Depressive Symptoms in the Adult Population in China: Single-Blind, Randomized Controlled Trial

**DOI:** 10.2196/55583

**Published:** 2025-02-13

**Authors:** Tingfei Zhu, Liuyi Zhang, Wenqi Weng, Ruochen Gan, Limin Sun, Yanping Wei, Yueping Zhu, Hongyan Yu, Jiang Xue, Shulin Chen

**Affiliations:** 1 Counseling and Psychological Services Shanghai Jiao Tong University Shanghai China; 2 Department of Psychology and Behavioral Sciences Zhejiang University Hangzhou China; 3 Department of Physical Education Shanghai Jiao Tong University Shanghai China; 4 The Affiliated Dongguan Songshan Lake Central Hospital Guangdong Medical University Dongguan China; 5 School of Humanities and Management Guangdong Medical University Dongguan China

**Keywords:** internet-based, self-guided, short-term, mindfulness, depression, randomized controlled trial

## Abstract

**Background:**

Depression is a significant global public health issue, and in China, access to mental health services remains limited despite high demand. Research has shown that mindfulness can effectively alleviate depressive symptoms and that telehealth solutions offer a promising avenue for addressing this service gap. Despite this potential, there are currently few studies in China focusing on short-term online mindfulness training. Most existing online mindfulness studies relied on traditional 8-week programs, which can be challenging for participant adherence due to limited accessibility and high dropout rates. Additionally, limited research exists on short-term online mindfulness interventions, and findings remain inconsistent.

**Objective:**

This study aimed to develop and evaluate an internet-based, self-guided, short-term mindfulness training (ISSMT) program based on the Monitor and Acceptance Theory (MAT) to reduce depression symptoms.

**Methods:**

The ISSMT program was delivered via an online platform, “Hi Emotion,” and was accessible to the general public. Interested individuals aged 18 years and older were randomized into either the ISSMT group or a wait-list control group. Participants in the ISSMT group received daily reminders to participate in a 15- to 20-minute session over a 14-day training period. Measurements, including mindfulness and depressive symptoms, were collected at baseline and weekly for the subsequent 3 weeks.

**Results:**

A total of 205 adults participated in the 14-day online intervention. Linear mixed models were used to analyze both per-protocol (PP) and intention-to-treat (ITT) samples. Compared with the wait-list control group, participants in the ISSMT group showed significant improvements in mindfulness (Cohen *d*=0.44 for ITT; Cohen *d*=0.55 for PP) and reductions in depressive symptoms (Cohen *d*=0.50 for ITT; Cohen *d*=0.53 for PP). Furthermore, participants expressed high acceptance of this training format with a relatively low dropout rate (<40%).

**Conclusions:**

The ISSMT program based on the MAT effectively enhanced mindfulness and alleviated depressive symptoms. This intervention could be considered for integration into psychosocial service systems to improve mental health outcomes and help bridge the gap between limited resources and the high demand for services in China. Future research should focus on personalizing these programs and incorporating advanced technologies to enhance their effectiveness and user engagement.

**Trial Registration:**

Open Science Framework; https://doi.org/10.17605/OSF.IO/8P4V6

## Introduction

Depression is a leading global public health issue, with a large impact on people’s well-being and quality of life and causing substantial economic ramifications and mental health–related disability [1]. According to the World Health Organization, approximately 280 million people worldwide, representing 5% of the adult population, grapple with depression [2,3]. The China Mental Health Survey reported that the weighted prevalence of depressive disorders in China is 3.6% (95% CI 3.0%-4.2%) during the previous 12 months and the lifetime weighted prevalence was 6.8% (95% CI 5.8%-7.8%) [4].

Given the considerable demand for mental health services, access to mental health services in China is limited. As of 2022, there were only 2098 psychiatric hospitals in China that were staffed by approximately 273,000 professionals, resulting in an average of 3.67 professionals serving 100,000 people [5]. Compounding this issue is the pervasive stigma associated with seeking mental health services. A recent study revealed that, in China, fewer than 10% of patients with depression received any form of treatment, with only 0.5% receiving adequate care [6].

The emergence of telehealth solutions has shown promise in bridging the mental health gap, especially since the COVID-19 pandemic. Telehealth solutions offer several key advantages including high acceptability both as a supplement and alternative to offline face-to-face training. A survey of 500 adults revealed a high interest (71.2%) in internet-based psychological training, with 42% expressing a preference for internet-based training over individual training (37.8%) and group training (19.6%) [7]. Telehealth solutions also provide a high level of accessibility, and users have the flexibility to use medical services according to their own schedule. In addition, telehealth offers greater anonymity, which is valuable for managing sensitive information and providing a more comfortable, private environment for users [8]. Compared with face-to-face training, the development and maintenance of online health care require relatively fewer economic and human resources [9]. Finally, several studies have indicated that some evidence-based psychological training can be effective in an online format, significantly ameliorating users’ depression and anxiety. The effect size (*d*=0.80) of self-help training protocols is only slightly lower than that of standardized treatment protocols (*d*=0.82) [10-15].

Mindfulness-based training has gained increasing attention in recent years [16-20], with substantial evidence supporting its positive impact on depression [19,20]. Mindfulness is a moment-by-moment awareness of thoughts, feelings, bodily sensations, and the surrounding environment [21]. Among the various mindfulness-based interventions, 2 of the most widely implemented are Mindfulness-Based Stress Reduction (MBSR) and mindfulness-based cognitive therapy [22]. MBSR, developed by Kabat-Zinn [23], and mindfulness-based cognitive therapy, created by Teasdale et al [24] as an extension of MBSR, are central to mindfulness practice. Both interventions typically include 8 weekly sessions focused on mindfulness techniques. A meta-analysis of 44 meta-analyses encompassing various populations, problems, training, comparisons, and outcomes revealed that mindfulness training demonstrated significant effects and advantages (*d*=0.10-0.89), including for depression (*d*=0.49), compared with negative controls [20].

The theoretical mechanisms underlying mindfulness interventions are supported by several key frameworks, including the Monitor and Acceptance Theory (MAT) [25], Mindfulness-to-Meaning Theory [26,27], and self-awareness, self-regulation, and self-transcendence framework [28]. Among these frameworks, MAT posits that attention monitoring and acceptance are essential mechanisms of mindfulness [25]. This dual-process approach enhances individuals’ ability to monitor internal experiences and accept them without judgment, which is fundamental to reducing negative emotions and fostering psychological flexibility, a key function in emotional regulation.

Compared with offline training, online mindfulness programs generally require less direct involvement from highly trained therapists, reducing reliance on professionals and enhancing accessibility [22]. Some studies have shown that online mindfulness programs, when matched for duration and structure, have slightly weaker effects than offline versions but still show significantly greater improvements than a control group [29]. Furthermore, the efficacy of online programs for alleviating depression has been supported by research in some western countries [15,30].

Most online mindfulness training programs are adaptations of traditional 8-week offline programs [31]. Although traditional programs have demonstrated efficacy, their extended duration and demanding requirements often result in challenges such as limited accessibility [31] and high dropout rates [32]. The few studies exploring the effect of simplified, short-term online mindfulness programs have shown inconsistent [33-36] and unstable effects [37,38]. Some studies with 2- to 4-week online mindfulness training reported positive results [33-35], while others, such as the 2-week self-help program evaluated by Glück and Maercker [36], found no significant differences between the mindfulness group and wait-list control group in terms of mindfulness scores, stress perception, overall distress, and more. To our knowledge, the feasibility and effectiveness of brief online mindfulness training in the Chinese population have rarely been investigated [31].

Current brief online mindfulness interventions often emphasize basic mindfulness techniques [39], primarily attention monitoring. MAT highlights the importance of incorporating acceptance, which is crucial for modulating emotional reactivity and stress responses, ultimately improving mental and physical well-being [25]. Interventions based on MAT may potentially address the limitations of existing internet-delivered programs by integrating both attention monitoring and acceptance. Randomized controlled trials (RCTs) have demonstrated that MAT-based training significantly improves stress-related indicators, including reductions in cortisol levels and systolic blood pressure reactivity [40], while also enhancing positive emotions [41].

The primary aim of this study was to implement an internet-based, self-guided, short-term mindfulness training (ISSMT) program based on MAT via a mobile platform with a Chinese population and to verify its effectiveness for depressive symptoms using an RCT design. We hypothesized that the ISSMT could be effectively delivered online and would lead to significant improvements in participants’ depressive symptoms.

## Methods

This study was designed as a single-blind RCT.

### Recruitment

A power analysis was conducted to estimate the sample size needed to detect the main effects and interactions in this study. Based on an effect size of 0.3 [20], a power of 0.80, and an alpha of .05, the initial calculation suggested that 126 participants would be required. Some researchers have reported high dropout rates with online training. For instance, an online MoodGym study reported a dropout rate of 73.9% for research participants and 99.2% for public registrants [42], and completion rates for internet-based depression treatment programs have varied from 43% to 99% [43]. To account for a potential dropout rate of 50%, which is commonly observed in online interventions, we planned to recruit 250 participants.

From January 2021 to April 2021, participants were recruited by posting recruitment posters on various public social media platforms, including WeChat Moment, RED app, Douban Forum, and other online forums. Interested individuals were directed to an online questionnaire, where they could register by scanning the QR code on the recruitment poster and providing basic demographic information. A total of 328 participants voluntarily enrolled in this study.

The inclusion criteria were as follows: (1) aged 18 years or older; (2) possessed intact reading and comprehension abilities; (3) had the capacity to provide informed consent; and (4) had access to the internet and basic smartphone literacy. Exclusion criteria included (1) current use of psychotropic medications, (2) severe visual and auditory impairments that hindered the comprehension of audio content or receipt of information, and (3) concurrent participation in other psychosocial intervention programs.

To enhance data representativeness, recruitment efforts spanned multiple platforms and were conducted at different times of the day to capture a diverse participant pool. Additionally, we used the neutral phrase “Peace Journey” in recruitment materials to mitigate self-selection bias.

Finally, 81 individuals who were unreachable after registration were excluded, leaving 247 eligible participants enrolled for randomization and the subsequent intervention.

### Procedure

After the initial registration, potential participants were invited to join a WeChat group for further communication. Preliminary screening was conducted based on the predefined inclusion and exclusion criteria. Eligible participants were invited to participate in subsequent research, were informed of the entire research process, and signed an electronic informed consent form. Baseline data were collected after receiving the informed consent form.

The unit of randomization in this study was the individual. Using computer-generated random numbers, we randomly assigned all eligible participants to either the ISSMT group or the wait-list control group. A statistician generated the randomization sequence, and baseline data were collected after the randomization. Investigators remained blinded to the randomization process. Notably, the investigation of online mindfulness training by Krusche et al [34] revealed a dropout rate of 79.4% for the training group, contrasting with only 35.9% for the wait-list control group. To balance the retained sample size in the 2 arms and adhere to statistical standards, we randomly assigned participants to groups at a ratio of 2.5:1 during randomization.

Ultimately, 175 participants were allocated to the ISSMT group, while 72 were assigned to the wait-list control group. Those in the control group received the same mindfulness training after completion of the formal study.

### Intervention

#### Training Course

In this study, the ISSMT program was translated and adapted from a 14-day mindfulness training program developed based on MAT by Lindsay et al [25]. With authorization from Lindsay and colleagues, a team of 4 psychology graduate students was assembled to perform the translation and adaptation. Each unit underwent a standardized multistep process, including sequential stages of “translation, peer cross-check, revision, group discussion and proofreading, and revision,” culminating in the creation of a draft. Team members then rotated assigned sections and completed a secondary round of proofreading. Following this, an expert in psychology was invited to conduct a comprehensive proofread of the entire text. Any segments identified as unclear were further refined in alignment with the original text until all team members reached consensus on clarity and accuracy.

The draft was subsequently recorded by a mindfulness practitioner with over 5 years of experience. We then invited 30 individuals to listen to the recordings and provide feedback, which was used to refine the audio content to finalize the training course. The finalized training framework, with each section lasting approximately 15 minutes to 20 minutes, is shown in Table 1.

**Table 1:** Lesson content in each training program based on the Monitor and Acceptance Theory (MAT).

Lesson	Content
1	Introduction: introduction to the course and 3 core skills: concentration, equanimity, and clarity
2	Concentration I: developing a deeper understanding of concentration
3	Concentration II: concentrating continuously on body experiences
4	Concentration III: maintaining focus on body experiences while listening to someone speak
5	Concentration IV: labeling body experiences to maintain focus
6	Concentration V: labeling different types of body experiences
7	Equanimity I: maintaining global body relaxation to promote equanimity
8	Equanimity II: promoting equanimity by intentionally using a matter-of-fact tone of voice when labeling
9	Clarity I: discriminating different types and patterns of body sensations
10	Clarity II: detecting subtle or faint body sensations and increasing sensual fulfillment by detecting subtle pleasure
11	Equanimity III: developing equanimity by applying a welcoming attitude toward all experiences
12	Clarity III: recognizing 4 basic categories of body experience (physical, emotional, restful, and “energy flow”)
13	Equanimity IV: integrating the 3 equanimity strategies: body relaxation, tone of voice, and welcoming attitude
14	Course Review: guided practice through the major strategies learned in the preceding 13 lessons

#### Intervention Platform

An online training platform, named “Hi Emotion,” was developed to facilitate the intervention. To access the training course, participants were only required to navigate to the “Hi Emotion” module within the “Mindfulness + Technology” section on WeChat. The platform accommodates 2 roles: “leader” and “practitioner” (see Figure S1A in Multimedia Appendix 1). The leader’s role involves creating groups and administering online courses, while practitioners have the option to join specific groups via the “Group” section. Once the “leader” disseminates the daily course, it becomes immediately visible to the “practitioner” in the “Unlearned Courses” section. Upon completion, the course is removed from the “Unlearned Courses” and relocated to the “Learned Courses” section.

#### Intervention Procedure

Following the randomization process, the researcher, acting as the “leader,” established a training group and distributed the group code to participants allocated to the ISSMT group. These participants then joined the group and familiarized themselves with the platform using the provided instruction manual. During the 14-day training period, the course was delivered daily on the platform at 10 AM. Participants received immediate reminders in the WeChat group after each course delivery, with additional reminders sent at 8 PM each evening.

The training lasted for 14 days, with each individual course lasting between 15 minutes and 20 minutes. Upon completion of the daily training course, participants were required to engage in mindfulness meditation practices outside the online course for a minimum of 30 minutes per day. In addition, participants were encouraged to incorporate mindfulness practices into their daily activities, such as during meals, while standing, and during walking.

To minimize the dropout rate, several measures were implemented. During the intervention, a trained experimenter sent timely course reminders in the WeChat group every day (at 10 AM and 8 PM) and addressed any participant queries. On the 4th and 10th days of the training, the researchers monitored the participants’ progress through the backend database. If any participants exhibited signs of potential dropout (such as incomplete listening records), the experimenter would contact them via WeChat to ensure they understood the course material and to encourage their continued participation.

### Measures

#### Demographic Characteristics

Demographic characteristics were assessed solely at baseline and encompassed age, gender, educational level, and occupation.

#### Mindfulness

The short form of the Chinese version of the 5-facet mindfulness questionnaire (FFMQ-C-SF) was used to measure mindfulness [44]. This 15-item scale measures 5 aspects of mindfulness on a 5-point Likert scale, ranging from 1 (never or very rarely true) to 5 (very often or always true), with higher total scores indicating a higher level of mindfulness. The Cronbach α for the FFMQ-C-SF in this study was 0.84.

#### Depressive Symptoms

Depressive symptoms were evaluated using the Beck Depression Inventory-II (BDI-II) [45]. This 21-item scale is scored on a scale from 0 to 3 according to the severity of symptoms and is one of the most widely used self-rated scales for depressive symptoms. It is applicable to the assessment of depressive symptoms in various clinical and nonclinical populations, with a total score of 0 to 13 indicating no depression, 14 to 19 indicating mild depression, 20 to 28 indicating moderate depression, and 29 to 63 indicating severe depression. The Chinese version of BDI-II, revised by Wang et al [46], demonstrated good reliability and validity, with a Cronbach α coefficient of 0.94. The Cronbach α coefficient for BDI-II in this study was 0.95.

#### Feasibility

The Intervention Satisfaction Scale designed by Campo et al [47] was used to measure the feasibility of participants’ participation and evaluated satisfaction with the internet-based training. It includes 9 items. Items 1 (“Overall, I liked the two-week course practice”) and 2 (“I would recommend this course to others”) are scored on a scale from 1 to 5 (1=strongly disagree, 5=strongly agree), while the remaining 7 items are scored on a 2-point scale (1=yes, 2=no).

### Data Quality Control

To ensure data quality and reliability throughout the study, we implemented multiple control measures. Standardized protocols were followed during data collection to reduce variability. During data cleaning, responses were rigorously screened, and entries displaying excessive duplication or failing to pass attention-check items were excluded. Specifically, attention checks included prompts such as “Please select the option ‘Moderately agree’ for this question,” allowing us to effectively identify inattentive respondents. During the data collection phase, we regularly monitored participants’ completion status and their engagement with the intervention (see the Intervention Procedure section for specifics) to maintain data integrity. These comprehensive monitoring and engagement strategies ensured data integrity and minimized the risk of dropout, enhancing the robustness and reliability of our study findings.

### Statistical Analysis

Descriptive statistics summarized participants’ demographic information and baseline outcome variables across different groups. To assess group differences, we used *t* tests for continuous variables and chi-square tests for categorical variables.

To analyze training effects in this longitudinal study, we used linear mixed models [48,49]. This approach accounts for both random and fixed effects, making it well-suited to handle missing data, which we assumed to be missing at random [50]. Specifically, compound symmetric covariance and maximum likelihood estimation were used.

We implemented both intention-to-treat (ITT) and per-protocol (PP) analyses [51]. ITT analysis includes all randomized participants, maintaining the benefits of randomization regardless of treatment adherence. PP analysis includes participants who completed the assigned treatment. In this study, the ITT analysis included participants who completed baseline measurements and participated in at least one course. The PP analysis focused on the participants who completed at least 75% of their assigned courses (ie, at least 10 sessions), which this study defined as completion of training. This approach enables a clear assessment of the actual treatment impact. Together, these methods provide a comprehensive view of the intervention’s effectiveness, with ITT generally yielding more conservative estimates.

Additionally, we evaluated the following effects: the time effect captures changes in measured outcomes across different time points, reflecting how participants’ performance evolved over the training duration. The group effect indicates differences in outcomes between treatment and control groups, illustrating the intervention’s impact. Finally, the time × group interaction examines how changes over time may differ across groups, elucidating the differential effectiveness of the intervention [52].

All analyses were conducted using SPSS 20.0 (IBM Corp) and incorporated age, gender, and other baseline variables as covariates. Statistical significance was determined using 2-sided tests, with a threshold of *P*<.05.

### Ethical Considerations

#### Ethics Approval

The study was approved by the Research Ethics Committee of the Department of Psychology and Behavioral Sciences, Zhejiang University (register number: 2020-059).

All procedures performed were in accordance with the ethical standards of the Zhejiang University research committee and with the 1964 Helsinki declaration and its later amendments or comparable ethical standards.

#### Informed Consent

Informed consent was obtained online from all participants before their involvement in the study.

#### Privacy and Confidentiality

Although the study involved repeated measurements from the same participants, the data collected were anonymized for analysis and feedback purposes. No personally identifiable information was included in the data nor the personalized feedback reports provided to participants.

#### Compensation Details

Participants received personalized feedback reports containing tailored insights based on their responses as compensation for their participation.

## Results

### Demographic Characteristics

**Figure 1:**
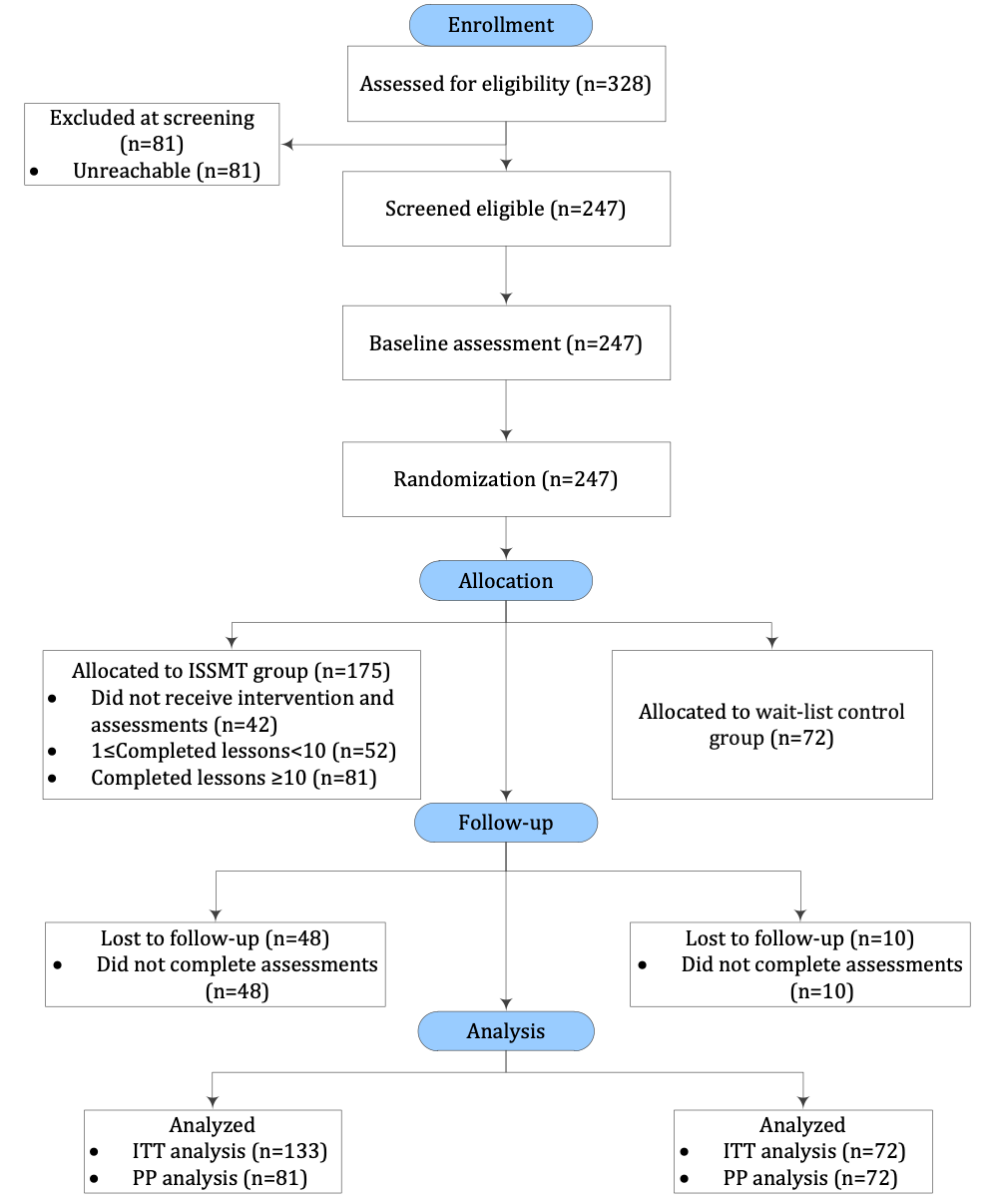
Research procedures. ISSMT: internet-based, self-guided, short-term mindfulness training; ITT: intention-to-treat; PP: per-protocol.

A total of 328 individuals voluntarily signed up to participate. Of these 328 individuals, 247 (75.3%) met the inclusion criteria and were randomized into the study. Data from 42 people who did not engage in any subsequent courses were excluded, leaving 205 eligible participants (133 in the training group and 72 in the wait-list control group). For the purposes of this study, individuals who completed more than 75% of the courses (ie, participation in at least 10 courses) were considered to have completed the training. A total of 81 participants (81/131, 60.9%) in the training group met this criterion (see Figure 1). No adverse events were reported during the study. Finally, data from 133 individuals in the training group and 72 individuals in the wait-list control group were included in the ITT analysis, while data from 81 and 72 participants, respectively, were included in the PP analysis.

The basic demographic information of the participants is shown in Table 2. The average ages of the participants in the training group and wait-list control group were 24.80 (SD 6.31) years and 24.60 (SD 6.62) years, respectively, with no statistically significant difference. There were also no significant differences between the 2 groups in terms of gender, education, or baseline mindfulness and depressive symptoms.

**Table 2:** Demographic characteristics (N=205).

Demographics characteristics			ISSMT^a^ group (n=133)		Wait-list control group (n=72)		Statistic (*df*)			*P* value	
Age (years), mean (SD)			24.8 (6.31)		24.6 (6.62)		0.21 (203)^b^			.83	
Female gender, n (%)			98 (73.7)		56 (77.8)		0.42 (1)^c^			.52	
**Education (years), n (%)**								2.67 (2)^c^			.26
	<12	5 (3.8)		3 (4.2)							
	12	70 (52.6)		46 (63.9)							
	>12	58 (43.6)		23 (31.9)							
Baseline FFMQ-C-SF^d^ score, mean (SD)			43.98 (8.04)		43.54 (8.92)		0.36 (203)^b^			.72	
Baseline BDI-II^e^ score, mean (SD)			12.81 (11.26)		14.84 (10.63)		–1.24 (203)^b^			.22	

^a^ISSMT: internet-based, self-guided, short-term mindfulness training.

^b^*t* test.

^c^Chi-square test.

^d^FFMQ-C-SF: short form of the Chinese version of the 5-facet mindfulness questionnaire.

^e^BDI-II: Beck Depression Inventory-II.

### Training Effects on Mindfulness

According to the ITT analysis, the scores for each outcome variable at each measurement time point are presented in Table 3. At baseline, there was no significant difference in mindfulness scores between the training group and the wait-list control group. The results of the mixed linear model indicated significant main effects of time (*F*_3,515.19_=29.48; *P*<.001), group (*F*_1,214.78_=10.20; *P*=.002), and the time × group interaction (*F*_3,515.25_=7.05; *P*<.001). The results suggest that the online mindfulness training significantly improved the participants’ mindfulness. The effect size for the change in mindfulness level was 0.44.

**Figure 2:**
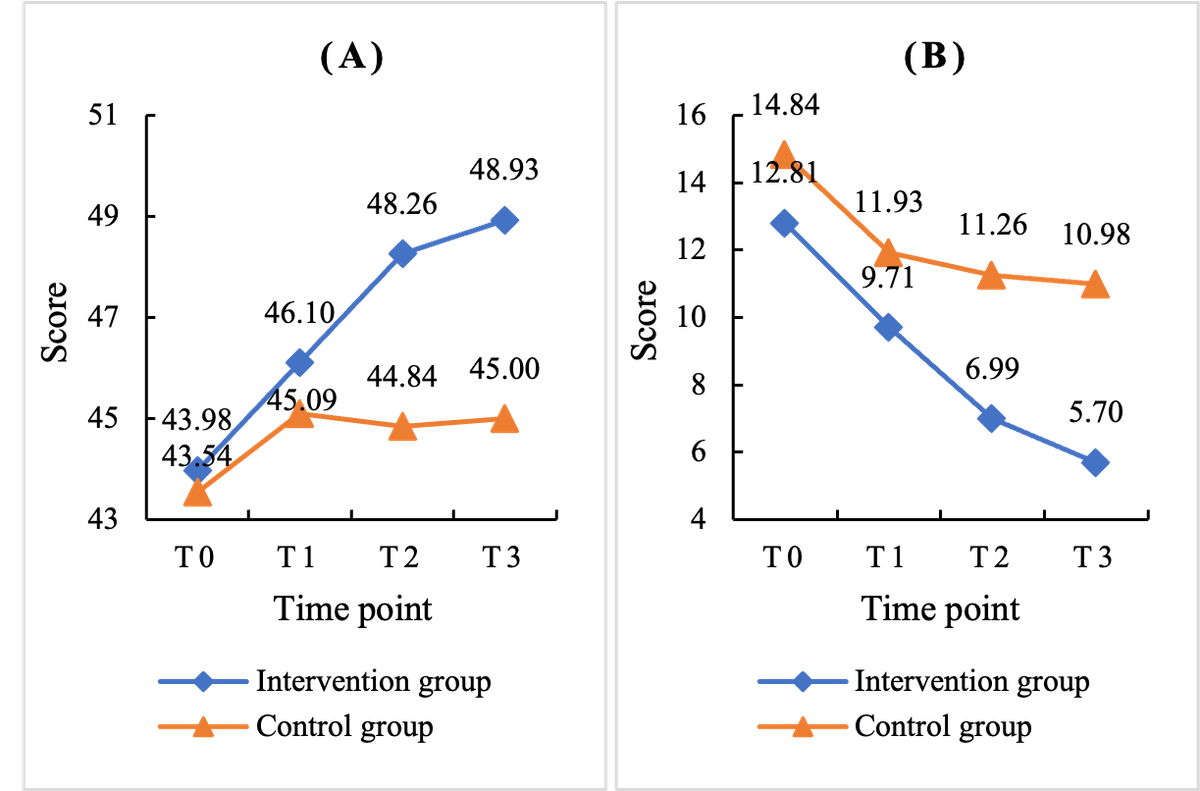
Intention-to-treat analysis of the comparison of (A) mindfulness, as measured using the short form of the Chinese version of the 5-facet mindfulness questionnaire (FFMQ-C-SF), and (B) depression, as measured using the Beck Depression Inventory-II (BDI-II), between the training and wait-list control groups at each time point.

As shown in Table 3, the training group demonstrated a significant increase in mindfulness starting from the first week, with continued significant improvements thereafter, whereas no significant changes were observed in the wait-list control group. The trends in mindfulness level changes at each time point are illustrated in Figure 2A.

According to the PP analysis, the scores for each outcome variable at each measurement time point are presented in Table 4. At baseline, there was no significant difference in mindfulness scores between the training group and wait-list control group. The results of the mixed linear model revealed significant main effects of time (*F*_3,419.57_ =29.31; *P*<.001), group (*F*_1,158.47_=11.21; *P*=.001), and the time × group interaction (*F*_3,419.64_=7.67; *P*<.001). These results indicate that the online mindfulness training significantly improved the participants’ mindfulness level. The effect size for the change in mindfulness level was 0.55.

**Figure 3:**
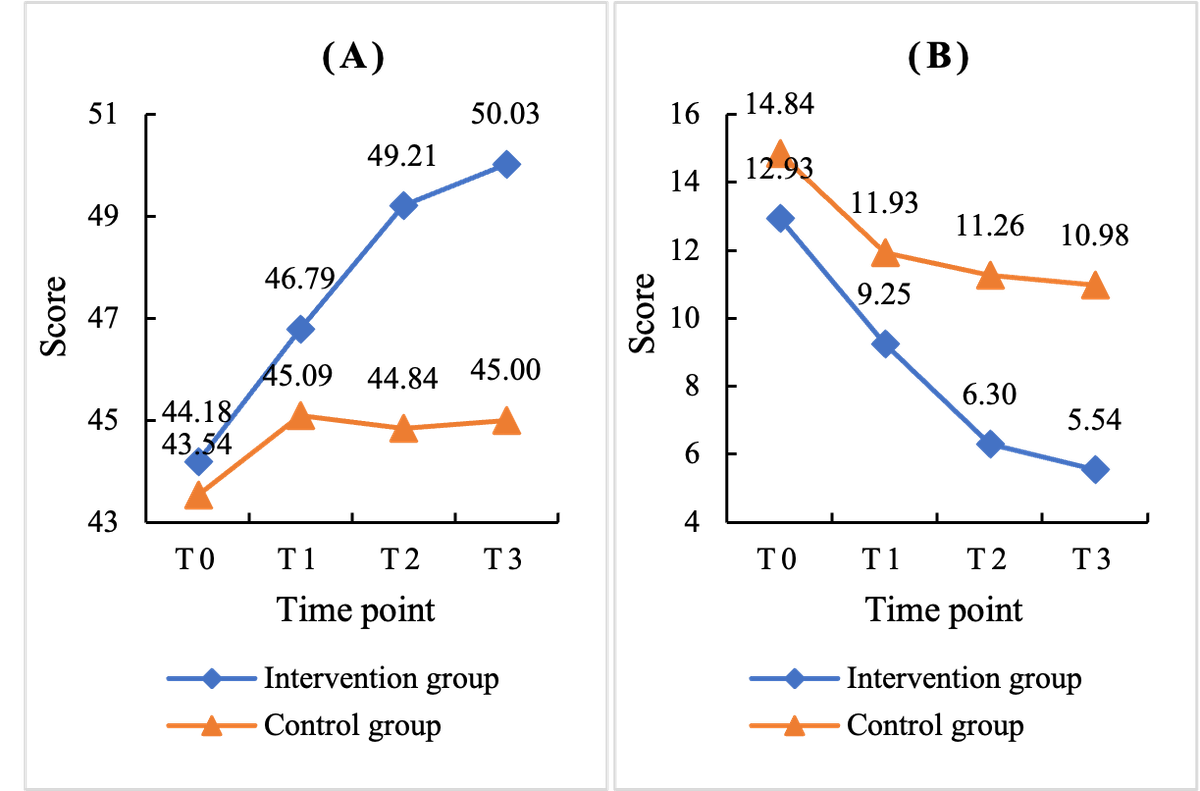
Per-protocol analysis of the comparison of (A) mindfulness, as measured using the short form of the Chinese version of the 5-facet mindfulness questionnaire (FFMQ-C-SF), and (B) depression, as measured using the Beck Depression Inventory-II (BDI-II), between the training and wait-list control groups at each time point.

As shown in Table 4, the mindfulness level in the training group showed a marginally significant increase starting from the first week and continued to increase significantly thereafter, while no significant changes were observed in the wait-list control group. The trends in mindfulness level changes at each time point are depicted in Figure 3A.

**Table 3:** Intention-to-treat analysis of the improvements in mindfulness and depression after online mindfulness training.

Time point			ISSMT^a^ group (n=133), mean (SD)		Wait-list control group (n=72), mean (SD)		*t* (*df*)		*P* value		Time effect				Group effect				Time × group interaction					Effect size (Cohen *d*)
											*F* (*df*)		*P* value		*F* (*df*)		*P* value		*F* (*df*)		*P* value			
**FFMQ-C-SF^b^**																								
	T_0_^c^	43.98 (8.04)		43.54 (8.92)		0.36 (203)		.72		—^d^		—		—		—		—		—		—		
	T_1_^e^	46.09 (8.25)		45.09 (9.18)		0.74 (173)		.46		—		—		—		—		—		—		—		
	T_2_^f^	48.26 (8.19)		44.84 (9.25)		2.41 (152)		.017		—		—		—		—		—		—		—		
	T_3_^g^	48.93 (8.08)		45 (9.75)		2.67 (146)		.008		29.48 (3, 515.19)		<.001		10.20 (1, 214.78)		.002		7.05 (3, 515.25)		<.001		0.44		
**BDI-II^h^**																								
	T_0_	12.81 (11.26)		14.84 (10.63)		–1.24 (203)		.22		—		—		—		—		—		—		—		
	T_1_	9.72 (10.02)		11.93 (11.24)		–1.33 (173)		.18		—		—		—		—		—		—		—		
	T_2_	6.99 (9.55)		11.26 (12.04)		–2.33 (107.71)		.02		—		—		—		—		—		—		—		
	T_3_	5.70 (7.82)		10.98 (12.78)		–2.89 (93.50)		.005		38.48 (3, 514.77)		<.001		9.31 (1, 207.10)		0.003		3.87 (3, 514.87)		.009		0.50		

^a^ISSMT: internet-based, self-guided, short-term mindfulness training.

^b^FFMQ-C-SF: short form of the Chinese version of the 5-facet mindfulness questionnaire.

^c^T_0_: baseline.

^d^Not applicable.

^e^T_1_: 1st week.

^f^T_2_: 2nd week.

^g^T_3_: 3rd week.

^h^BDI-II: Beck Depression Inventory-II.

**Table 4:** Per-protocol analysis of the improvements in mindfulness and depression after online mindfulness training.

Time period		ISSMT^a^ group (n=81), mean (SD)	Wait-list control group (n=72), mean (SD)	*t* (*df*)	*P* value	Time effect			Group effect			Time × group interaction			Effect size (Cohen *d*)
						*F* (*df*)	*P* value	*F* (*df*)		*P* value	*F* (*df*)		*P* value		
**FFMQ-C-SF^b^**															
	T_0_^c^	44.18 (8.89)	43.54 (8.92)	0.43 (151)	.67	—^d^	—	—		—	—		—	—	
	T_1_^e^	46.79 (8.58)	45.09 (9.18)	1.14 (144)	.25	—	—	—		—	—		—	—	
	T_2_^f^	49.21 (8.32)	44.84 (9.25)	2.86 (130)	.005	—	—	—		—	—		—	—	
	T_3_^g^	50.03 (8.59)	45.00 (9.75)	3.09 (125)	.002	29.31 (3, 419.57)	<.001	11.21 (1, 158.47)		.001	7.67 (3, 419.64)		<.001	0.55	
**BDI-II^h^**															
	T_0_	12.93 (11.42)	14.84 (10.63)	–1.06 (151)	.29	—	—	—		—	—		—	—	
	T_1_	9.25 (8.66)	11.93 (11.24)	–1.60 (144)	.11	—	—	—		—	—		—	—	
	T_2_	6.30 (7.66)	11.26 (12.04)	–2.77 (98.76)	.007	—	—	—		—	—		—	—	
	T_3_	5.54 (7.15)	10.98 (12.78)	–2.94 (94.80)	.004	38.58 (3, 420.91)	<.001	10.11 (1, 157.77)		.002	3.65 (3, 421.03)		.01	0.53	

^a^ISSMT: internet-based, self-guided, short-term mindfulness training.

^b^FFMQ-C-SF: short form of the Chinese version of the 5-facet mindfulness questionnaire.

^c^T_0_: baseline.

^d^Not applicable.

^e^T_1_: 1st week.

^f^T_2_: 2nd week.

^g^T_3_: 3rd week.

^h^BDI-II: Beck Depression Inventory-II.

**Figure 5:**
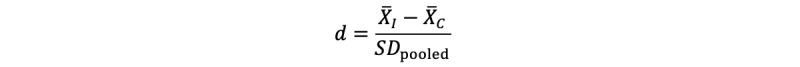
Inline graphic 2.

### Training Effects on Depressive Symptoms

According to the ITT analysis, the scores for each outcome variable at each measurement time point are presented in Table 3. At baseline, there was no significant difference in depression scores between the training group and wait-list control group. The results of the mixed linear model revealed significant effects of time (*F*_3,514.77_=38.48; *P*<.001), group (*F*_1,207.10_=9.31; *P*=.003), and the time × group interaction (*F*_3,514.87=_3.87; *P*=.009). These findings indicate that ISSMT can significantly reduce depressive symptoms, with an effect size of 0.50 for improving depression.

As shown in Table 3, depressive symptoms in the training group improved significantly from the first week and continued to improve significantly thereafter, whereas no significant changes were observed in the wait-list control group. The trends in the depression changes at each time point are illustrated in Figure 2B.

According to the PP analysis, the scores for each outcome variable at each time point are presented in Table 4. At baseline, there were no significant differences in depression scores between the training group and wait-list control group. The results of the mixed linear model revealed significant effects of time (*F*_3,420.91_=38.58; *P*<.001), group (*F*_1,157.77_=10.11; *P*=.002), and the time × group interaction (*F*_3,421.03_=3.65; *P*=.01). These findings indicate that online mindfulness training can significantly reduce depressive symptoms. The effect size for improving depression was 0.53.

As shown in Table 4, the depression score in the training group improved significantly from the first week and continued to improve significantly thereafter, while no significant changes were observed in the wait-list control group. The trends in the depressive symptom changes at each time point are shown in Figure 3B.

### Feasibility Analysis

The average Intervention Satisfaction Scale score for item 1 (“Overall, I liked the two-week course practice”) was 3.77, and the average score for item 2 (“I would recommend this course to others”) was 3.52, exceeding the average. At the same time, 87% (77/89) of the participants said they thought the course was helpful to them, and 72% (64/89) of the participants said they liked this online training method.

## Discussion

### Principal Findings

Depression is a leading global public health problem, with enormous demand for mental health services but limited access to mental health services. The emergence of telehealth solutions offers a promising approach to bridging this service gap. However, despite the potential, there are currently few studies in China that have focused on short-term online mindfulness training, with the majority of online studies adhering to the traditional 8-week mindfulness training framework [53]. In response, we developed a 2-week ISSMT course based on the MAT model. Additionally, we created an online platform for program delivery. The results from our RCT indicate that, compared with the wait-list control group, participants in the training group had increased mindfulness levels and decreased depressive symptoms. Furthermore, participants expressed high acceptance of this training format. This study provides empirical evidence supporting the feasibility and effectiveness of ISSMT for Chinese adults. We recommend that future research explore the integration of this type of training into psychosocial service systems to enhance mental health.

Although the effectiveness of traditional 8-week mindfulness training has been reported in some studies, concerns remain regarding the extended training duration. Scholars have noted that longer training periods may lead to higher dropout rates and reduced participant motivation, thereby affecting treatment adherence [32]. Adherence is a critical determinant of treatment outcome, with approximately 50% of treatment failures attributable to nonadherence [54]. In a study delivering online psychosocial interventions, high dropout rates have been observed, reaching up to 73.9% among research participants and as high as 99.2% among public registrants [42]. This study introduces an ISSMT program that can be delivered online and demonstrated a notably lower dropout rate (<40%) as well as effective implementation. We believe the adapted training program, customized delivery platform, and quality control measures used in this study can serve as a valuable reference for further practices in delivering online mindfulness training and other psychosocial interventions in China.

Although previous studies have reported inconsistent results for short-term online mindfulness training [34-36], our study found that ISSMT significantly reduced depressive symptoms in the general population. Specifically, no significant differences in depressive symptoms were observed between the 2 groups at baseline or at week 1. However, by weeks 2 and 3, the severity of depressive symptoms in the training group was significantly lower than that in the control group. Although previous research suggests that brief mindfulness meditation sessions can improve immediate emotional states (eg, a single 10-minute mindfulness meditation training can reduce the intensity of negative affect in response to emotional stimuli [55]), our findings indicated that certain “doses” of training may be necessary to ensure the effectiveness of internet-delivered training. Furthermore, the emphasis on acceptance in MAT may be a key factor for sustaining the intervention’s effects [25].

There are several strengths of the ISSMT program delivered in the study. First, the effect sizes for the improvement in mindfulness and reduction in depressive symptoms exceeded those reported in other similar studies [56], with these benefits maintained at follow-up. This suggests that some participants may have continued practicing mindfulness mediation in their daily life after the trial concluded, further supporting the feasibility and long-term potential of the ISSMT program for the Chinese population.

Moreover, the dropout rate in this study was lower than in other similar online training programs. High dropout rates are a well-documented challenge for internet-based psychological training, with reported rates ranging from 73.9% to 99.2% in other studies [42,43,57,58]. Another review reported that an additional 0% to 18% of participants were lost between the posttraining assessment and follow-up assessment [59]. In contrast, our study achieved a completion rate of 60.9%, reflecting a considerably lower dropout rate and confirming the acceptability of this training format.

China’s large internet user base underscores the considerable potential of digital health interventions on a global scale, particularly within significant internet economies like China. According to the 54th “Statistical Report on the Development of China’s Internet” released by the China Internet Network Information Center, as of August 2024, the number of internet users in China was 1.099 billion, with an internet penetration rate of 78% [60].

The convenience and accessibility of online training programs make them an important supplement to offline training, helping to bridge the gap between limited medical resources and the vast mental health service needs in China [61]. Despite a more than twofold increase in the national ratio of psychiatrists, including accredited and assistant psychiatrists, per 100,000 population from 2002 to 2020 (from 1.51 to 3.19) and for accredited psychiatrists specifically (from 1.20 to 2.89) [62], there remains a significant gap between the demand for mental health services and the available resources. This disparity is exacerbated by the uneven distribution of mental health professionals, with a concentration in major cities and the northeast and east regions of China [62]. In light of these challenges, this study developed and validated an online mindfulness training program that effectively overcomes the geographical limitations of traditional face-to-face interventions, underscoring its significance for areas with constrained medical resources.

From a policy perspective, the empirical evidence presented in this study offers a valuable benchmark for authorities and stakeholders when shaping mental health policies. Our results demonstrate that digital mental health interventions not only deliver effective therapeutic outcomes but also garner high user satisfaction. Based on our knowledge, China has begun pilot initiatives to incorporate psychological therapies into public health insurance in certain regions, including Guangdong, Jiangsu, and Beijing. Although traditional psychotherapy is known for its extended treatment periods and challenges in efficacy monitoring, this study shows that a 2-week intervention can yield significant benefits. This finding supports the proposition for policymakers to integrate comparable online intervention models into national or regional mental health frameworks. Integrating such models would enhance the provision of accessible and efficient mental health services to the population.

It is noteworthy that this study did not account for the effects of group-specific differences, leaving uncertainties whether the findings extend to high-risk populations for depression, such as older adults and pregnant women. Older adults may face unique physiological and psychological challenges, including cognitive decline and difficulties with technology use [63], that may influence their acceptance of and response to online interventions. For pregnant women, prior research has shown that those with high levels of neuroticism demonstrate greater improvements in maladaptive cognitive emotion regulation strategies following mindfulness interventions compared with those with moderate or low neuroticism [64]. This suggests variability in response to mindfulness interventions based on neuroticism levels. Therefore, caution is advised when generalizing these findings to these specific populations.

### Limitations

A couple of limitations should be mentioned for this study. First, the main intervention effects were based primarily on self-report data. Further research may combine a broader range of measures, such as behavioral and biological measures. Second, a wait-list control design was used, and using an active control intervention in future studies would provide stronger support for the specific effects of the program. Additionally, this study did not segment participants by specific demographic categories. Certain groups, such as older adults and pregnant women, are recognized as higher-risk populations for depression, and the absence of targeted analysis for these groups may limit the generalizability of our findings. Future research should consider implementing strategies to increase the inclusion of high-risk groups, ensuring a broader and more diverse sample. For instance, offline recruitment methods or targeted online advertisements could be used to reach these specific populations. These enhancements would strengthen the representativeness of the sample and improve the external validity of the findings.

### Conclusions

The 2-week ISSMT course based on the MAT demonstrated high feasibility and effectiveness for enhancing mindfulness levels and alleviating depressive symptoms. Given China’s substantial potential user base and the disparity between limited mental health resources and the significant demand for services, this digital health intervention could play a pivotal role in expanding access to mental health support. Future research should prioritize the development of personalized online mindfulness programs that consider individual differences such as age, gender, and daily emotional fluctuations. Integrating artificial intelligence [65], wearable technology, and diverse health data may further refine the precision and efficacy of these interventions. Additionally, exploring virtual reality [66] and natural language processing could enable more immersive and engaging user experiences, potentially enhancing therapeutic outcomes.

## Appendix

Multimedia Appendix 1 Example of the course platform interface: (A) leader and (B) practitioner.

Multimedia Appendix 2 CONSORT-eHEALTH checklist (V 1.6.1).

## Abbreviations

BDI-II: Beck Depression Inventory-II
FFMQ-C-SF: short form of the Chinese version of the 5-facet mindfulness questionnaire
ISSMT: internet-based, self-guided, short-term mindfulness training
ITT: intention-to-treat
MAT: Monitor and Acceptance Theory
MBSR: Mindfulness-Based Stress Reduction
PP: per-protocol
RCT: randomized controlled trial

## Data Availability

The data sets analyzed during this study are available from the corresponding author upon reasonable request.

## References

[1] Herrman H, Kieling C, McGorry P, Horton R, Sargent J, Patel V (2019). Reducing the global burden of depression: a Lancet-World Psychiatric Association Commission. Lancet.

[2] (2023). Depressive disorder (depression). World Health Organization.

[3] GBD Results. Institute for Health Metrics and Evaluation.

[4] Huang Y, Wang Y, Wang H, Liu Z, Yu X, Yan J, Yu Y, Kou C, Xu X, Lu J, Wang Z, He S, Xu Y, He Y, Li T, Guo W, Tian H, Xu G, Xu X, Ma Y, Wang L, Wang L, Yan Y, Wang B, Xiao S, Zhou L, Li L, Tan L, Zhang T, Ma C, Li Q, Ding H, Geng H, Jia F, Shi J, Wang S, Zhang N, Du X, Du X, Wu Y (2019). Prevalence of mental disorders in China: a cross-sectional epidemiological study. Lancet Psychiatry.

[5] (2022). China Statistical Yearbook 2022. National Bureau of Statistics China.

[6] Lu J, Xu X, Huang Y, Li T, Ma C, Xu G, Yin H, Xu X, Ma Y, Wang L, Huang Z, Yan Y, Wang B, Xiao S, Zhou L, Li L, Zhang Y, Chen H, Zhang T, Yan J, Ding H, Yu Y, Kou C, Shen Z, Jiang L, Wang Z, Sun X, Xu Y, He Y, Guo W, Jiang L, Li S, Pan W, Wu Y, Li G, Jia F, Shi J, Shen Z, Zhang N (2021). Prevalence of depressive disorders and treatment in China: a cross-sectional epidemiological study. Lancet Psychiatry.

[7] Wahbeh H, Svalina MN, Oken BS (2014). Group, one-on-one, or internet? Preferences for mindfulness meditation delivery format and their predictors. Open Med J.

[8] Ryan ML, Shochet IM, Stallman HM (2014). Universal online interventions might engage psychologically distressed university students who are unlikely to seek formal help. Advances in Mental Health.

[9] Elbert NJ, van Os-Medendorp H, van Renselaar W, Ekeland AG, Hakkaart-van Roijen L, Raat H, Nijsten TEC, Pasmans SGMA (2014). Effectiveness and cost-effectiveness of ehealth interventions in somatic diseases: a systematic review of systematic reviews and meta-analyses. J Med Internet Res.

[10] Berger T, Boettcher J, Caspar F (2014). Internet-based guided self-help for several anxiety disorders: a randomized controlled trial comparing a tailored with a standardized disorder-specific approach. Psychotherapy (Chic).

[11] Foroushani PS, Schneider J, Assareh N (2011). Meta-review of the effectiveness of computerised CBT in treating depression. BMC Psychiatry.

[12] Newby JM, Twomey C, Yuan Li SS, Andrews G (2016). Transdiagnostic computerised cognitive behavioural therapy for depression and anxiety: A systematic review and meta-analysis. J Affect Disord.

[13] Christ C, Schouten MJ, Blankers M, van Schaik DJ, Beekman AT, Wisman MA, Stikkelbroek YA, Dekker JJ (2020). Internet and computer-based cognitive behavioral therapy for anxiety and depression in adolescents and young adults: systematic review and meta-analysis. J Med Internet Res.

[14] Reger MA, Gahm GA (2009). A meta-analysis of the effects of internet- and computer-based cognitive-behavioral treatments for anxiety. J Clin Psychol.

[15] Spijkerman MPJ, Pots WTM, Bohlmeijer ET (2016). Effectiveness of online mindfulness-based interventions in improving mental health: A review and meta-analysis of randomised controlled trials. Clin Psychol Rev.

[16] Osborne EL, Ainsworth B, Hooper N, Atkinson MJ (2023). Experiences of using digital mindfulness-based interventions: rapid scoping review and thematic synthesis. J Med Internet Res.

[17] O'Brien-Venus B, Ellett L, Burgess-Barr S, Chadwick P (2024). Systematic review of the safety of mindfulness-based interventions for psychosis. Clin Psychol Rev.

[18] Yue WL, Ng KK, Koh AJ, Perini F, Doshi K, Zhou JH, Lim J (2023). Mindfulness-based therapy improves brain functional network reconfiguration efficiency. Transl Psychiatry.

[19] Goldberg SB, Tucker RP, Greene PA, Davidson RJ, Wampold BE, Kearney DJ, Simpson TL (2018). Mindfulness-based interventions for psychiatric disorders: A systematic review and meta-analysis. Clin Psychol Rev.

[20] Goldberg SB, Riordan KM, Sun S, Davidson RJ (2022). The empirical status of mindfulness-based interventions: a systematic review of 44 meta-analyses of randomized controlled trials. Perspect Psychol Sci.

[21] Zhang D, Lee EKP, Mak ECW, Ho CY, Wong SYS (2021). Mindfulness-based interventions: an overall review. Br Med Bull.

[22] Sommers-Spijkerman M, Austin J, Bohlmeijer E, Pots W (2021). New evidence in the booming field of online mindfulness: an updated meta-analysis of randomized controlled trials. JMIR Ment Health.

[23] Kabat-Zinn J (2003). Mindfulness-based stress reduction (MBSR). Constructivism in the Human Sciences.

[24] Teasdale JD, Segal ZV, Williams JM, Ridgeway VA, Soulsby JM, Lau MA (2000). Prevention of relapse/recurrence in major depression by mindfulness-based cognitive therapy. J Consult Clin Psychol.

[25] Lindsay EK, Creswell JD (2017). Mechanisms of mindfulness training: Monitor and Acceptance Theory (MAT). Clin Psychol Rev.

[26] Garland EL, Farb NA, Goldin P, Fredrickson BL (2015). Mindfulness broadens awareness and builds eudaimonic meaning: a process model of mindful positive emotion regulation. Psychol Inq.

[27] McConnell PA, Froeliger B (2015). Mindfulness, mechanisms and meaning: perspectives from the cognitive neuroscience of addiction. Psychol Inq.

[28] Vago DR, Silbersweig DA (2012). Self-awareness, self-regulation, and self-transcendence (S-ART): a framework for understanding the neurobiological mechanisms of mindfulness. Front Hum Neurosci.

[29] Compen F, Bisseling E, Schellekens M, Donders R, Carlson L, van der Lee M, Speckens A (2018). Face-to-face and internet-based mindfulness-based cognitive therapy compared with treatment as usual in reducing psychological distress in patients with cancer: a multicenter randomized controlled trial. JCO.

[30] Hearn JH, Cotter I, Finlay KA (2019). Efficacy of internet-delivered mindfulness for improving depression in caregivers of people with spinal cord injuries and chronic neuropathic pain: a randomized controlled feasibility trial. Arch Phys Med Rehabil.

[31] Jie G, Tong H, Sun X (2019). Effect of Chinese online mindfulness-based stress reduction course on mindfulness level and mood state of general population. Chinese General Practice.

[32] Fortney L, Luchterhand C, Zakletskaia L, Zgierska A, Rakel D (2013). Abbreviated mindfulness intervention for job satisfaction, quality of life, and compassion in primary care clinicians: a pilot study. Ann Fam Med.

[33] Kemper KJ (2017). Brief online mindfulness training: immediate impact. J Evid Based Complementary Altern Med.

[34] Krusche A, Cyhlarova E, Williams JMG (2013). Mindfulness online: an evaluation of the feasibility of a web-based mindfulness course for stress, anxiety and depression. BMJ Open.

[35] Cavanagh K, Strauss C, Cicconi F, Griffiths N, Wyper A, Jones F (2013). A randomised controlled trial of a brief online mindfulness-based intervention. Behav Res Ther.

[36] Glück TM, Maercker A (2011). A randomized controlled pilot study of a brief web-based mindfulness training. BMC Psychiatry.

[37] Zeidan F, Johnson SK, Diamond BJ, David Z, Goolkasian P (2010). Mindfulness meditation improves cognition: evidence of brief mental training. Conscious Cogn.

[38] Chen Y, Yang X, Wang L, Zhang X (2013). A randomized controlled trial of the effects of brief mindfulness meditation on anxiety symptoms and systolic blood pressure in Chinese nursing students. Nurse Educ Today.

[39] Howarth A, Smith JG, Perkins-Porras L, Ussher M (2019). Effects of brief mindfulness-based interventions on health-related outcomes: a systematic review. Mindfulness.

[40] Lindsay EK, Young S, Smyth JM, Brown KW, Creswell JD (2018). Acceptance lowers stress reactivity: Dismantling mindfulness training in a randomized controlled trial. Psychoneuroendocrinology.

[41] Lindsay EK, Chin B, Greco CM, Young S, Brown KW, Wright AGC, Smyth JM, Burkett D, Creswell JD (2018). How mindfulness training promotes positive emotions: dismantling acceptance skills training in two randomized controlled trials. J Pers Soc Psychol.

[42] Christensen H, Griffiths KM, Korten AE, Brittliffe K, Groves C (2004). A comparison of changes in anxiety and depression symptoms of spontaneous users and trial participants of a cognitive behavior therapy website. J Med Internet Res.

[43] Christensen H, Griffiths KM, Farrer L (2009). Adherence in internet interventions for anxiety and depression. J Med Internet Res.

[44] Zhu T, Chen C, Chen S (2021). Validation of a Chinese version of the five facet mindfulness questionnaire and development of a short form based on item response theory. Curr Psychol.

[45] Beck AT (1961). An inventory for measuring depression. Arch Gen Psychiatry.

[46] Wang Z, Yuan CM, Huang J, Li ZZ, Chen J, Zhang HY, Fang YR, Xiao ZP (2011). Reliability and validity of the Chinese version of Beck Depression Inventory-II among depression patients. Chinese Mental Health Journal.

[47] Campo RA, Bluth K, Santacroce SJ, Knapik S, Tan J, Gold S, Philips K, Gaylord S, Asher GN (2017). A mindful self-compassion videoconference intervention for nationally recruited posttreatment young adult cancer survivors: feasibility, acceptability, and psychosocial outcomes. Support Care Cancer.

[48] Tang W, He H, Tu XM (2012). Applied Categorical and Count Data Analysis.

[49] Kowalski J, Tu XM (2008). Modern Applied U‐Statistics.

[50] Lu N, Tang W, He H, Yu Q, Crits-Christoph P, Zhang H, Tu X (2009). On the impact of parametric assumptions and robust alternatives for longitudinal data analysis. Biom J.

[51] Fisher L, Polonsky W, Parkin CG, Jelsovsky Z, Amstutz L, Wagner RS (2011). The impact of blood glucose monitoring on depression and distress in insulin-naïve patients with type 2 diabetes. Curr Med Res Opin.

[52] Tabachnick B, Fidell L (2018). Using Multivariate Statistics, 7th edition.

[53] Mak WWS, Chan ATY, Cheung EYL, Lin CLY, Ngai KCS (2015). Enhancing web-based mindfulness training for mental health promotion with the health action process approach: randomized controlled trial. J Med Internet Res.

[54] Naderi SH, Bestwick JP, Wald DS (2012). Adherence to drugs that prevent cardiovascular disease: meta-analysis on 376,162 patients. Am J Med.

[55] Erisman SM, Roemer L (2010). A preliminary investigation of the effects of experimentally induced mindfulness on emotional responding to film clips. Emotion.

[56] Sverre KT, Nissen ER, Farver-Vestergaard I, Johannsen M, Zachariae R (2023). Comparing the efficacy of mindfulness-based therapy and cognitive-behavioral therapy for depression in head-to-head randomized controlled trials: a systematic review and meta-analysis of equivalence. Clin Psychol Rev.

[57] Christensen H, Griffiths KM, Mackinnon AJ, Brittliffe K (2006). Online randomized controlled trial of brief and full cognitive behaviour therapy for depression. Psychol Med.

[58] Farvolden P, Denisoff E, Selby P, Bagby RM, Rudy L (2005). Usage and longitudinal effectiveness of a web-based self-help cognitive behavioral therapy program for panic disorder. J Med Internet Res.

[59] Melville KM, Casey LM, Kavanagh DJ (2010). Dropout from internet-based treatment for psychological disorders. Br J Clin Psychol.

[60] CNNIC (2024). The 54th Statistical Report on Internet Development in China. China Internet Network Information Center.

[61] Kazdin AE, Rabbitt SM (2013). Novel models for delivering mental health services and reducing the burdens of mental illness. Clinical Psychological Science.

[62] Sun M, Zhou H, Li Y, Wang J, Yang W, Gong Y, Xu J, Zhang J, Yang X, Bueber M, Phillips MR, Zhou L (2024). Professional characteristics, numbers, distribution and training of China's mental health workforce from 2000 to 2020: a scoping review. Lancet Reg Health West Pac.

[63] Long KM, Casey K, Bhar S, Al Mahmud A, Curran S, Hunter K, Lim MH (2022). Understanding perspectives of older adults on the role of technology in the wider context of their social relationships. Ageing and Society.

[64] Wang Z, Zhang Q, Zhou Z, Huang Z, Mao H, Zhang L, Wang H (2023). Online mindfulness-based cognitive behavioral intervention for pregnant women: an efficacy evaluation. Maternal and Child Health Care of China.

[65] Deniz-Garcia A, Fabelo H, Rodriguez-Almeida AJ, Zamora-Zamorano G, Castro-Fernandez M, Alberiche Ruano MDP, Solvoll T, Granja C, Schopf TR, Callico GM, Soguero-Ruiz C, Wägner Ana M, WARIFA Consortium (2023). Quality, usability, and effectiveness of mHealth apps and the role of artificial intelligence: current scenario and challenges. J Med Internet Res.

[66] Pardini S, Gabrielli S, Olivetto S, Fusina F, Dianti M, Forti S, Lancini C, Novara C (2024). Personalized virtual reality compared with guided imagery for enhancing the impact of progressive muscle relaxation training: pilot randomized controlled trial. JMIR Ment Health.

