# Correction: Effectiveness of an Internet-Based, Self-Guided, Short-Term Mindfulness Training (ISSMT) Program for Relieving Depressive Symptoms in the Adult Population in China: Single-Blind, Randomized Controlled Trial

**DOI:** 10.2196/81689

**Published:** 2025-09-03

**Authors:** Tingfei Zhu, Liuyi Zhang, Wenqi Weng, Ruochen Gan, Limin Sun, Yanping Wei, Yueping Zhu, Hongyan Yu, Jiang Xue, Shulin Chen

**Affiliations:** 1 Counseling and Psychological Services Shanghai Jiao Tong University Shanghai China; 2 Department of Psychology and Behavioral Sciences Zhejiang University Hangzhou China; 3 Department of Physical Education Shanghai Jiao Tong University Shanghai China; 4 The Affiliated Dongguan Songshan Lake Central Hospital Guangdong Medical University Dongguan China; 5 School of Humanities and Management Guangdong Medical University Dongguan China

In “Effectiveness of an Internet-Based, Self-Guided, Short-Term Mindfulness Training (ISSMT) Program for Relieving Depressive Symptoms in the Adult Population in China: Single-Blind, Randomized Controlled Trial” (J Med Internet Res 2025;27:e55583) the authors noted one error.

The following statement was added to the author contribution section:

Jiang Xue should be considered a co‑corresponding author and can be contacted at jiang_xue@gdmu.edu.cn.

The correction will appear in the online version of the paper on the JMIR Publications website together with the publication of this correction notice. Because this was made after submission to PubMed, PubMed Central, and other full-text repositories, the corrected article has also been resubmitted to those repositories.

