# Supplementary figures and images for: Effectiveness of an Internet-Based, Self-Guided, Short-Term Mindfulness Training (ISSMT) Program for Relieving Depressive Symptoms in the Adult Population in China: Single-Blind, Randomized Controlled Trial
Source: J Med Internet Res. 2025 Feb 13;27:e55583. doi: 10.2196/55583 (PMC11888059; doi:10.2196/55583)

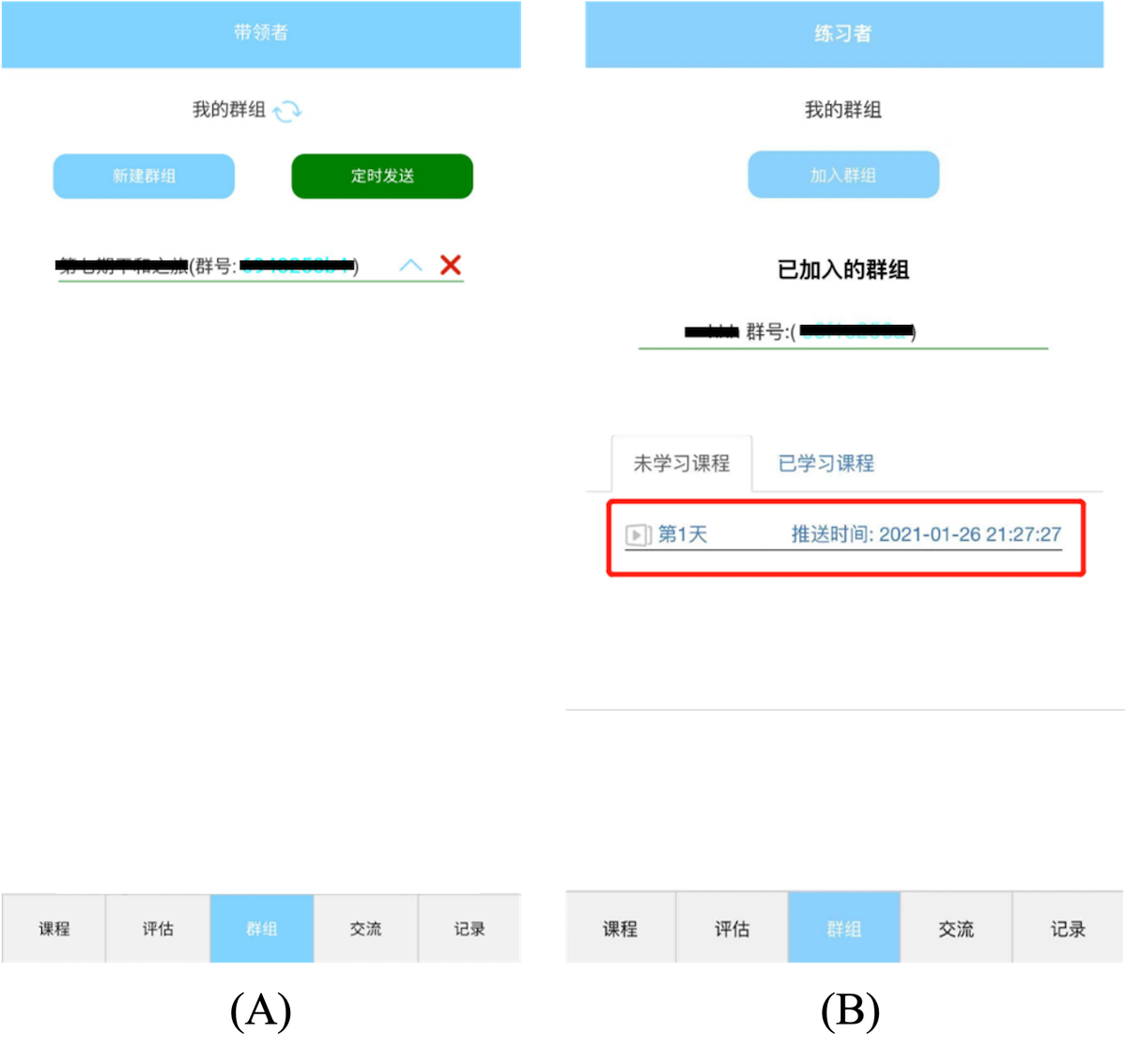

Supplement: Multimedia Appendix 1 [file jmir_v27i1e55583_app1.png]
